# Gene-set meta-analysis of lung cancer identifies pathway related to systemic lupus erythematosus

**DOI:** 10.1371/journal.pone.0173339

**Published:** 2017-03-08

**Authors:** Albert Rosenberger, Melanie Sohns, Stefanie Friedrichs, Rayjean J. Hung, Gord Fehringer, John McLaughlin, Christopher I. Amos, Paul Brennan, Angela Risch, Irene Brüske, Neil E. Caporaso, Maria Teresa Landi, David C. Christiani, Yongyue Wei, Heike Bickeböller

**Affiliations:** 1 Department of Genetic Epidemiology, University Medical Center, Georg-August-University Göttingen, Göttingen, Germany; 2 Lunenfeld-Tanenbaum Research Institute of Mount Sinai Hospital, Toronto, Canada; 3 Dalla Lana School of Public Health, University of Toronto, Toronto, Canada; 4 Public Health Ontario, Toronto, Canada; 5 Department of Biomedical Data Science, Geisel School of Medicine at Dartmouth, Hanover, New Hampshire, United States of America; 6 International Agency for Research on Cancer, Lyon, France; 7 Division of Molecular Biology, University Salzburg, Salzburg, Austria; 8 Institute of Epidemiology I, Helmholtz Center Munich, Munich, Germany; 9 Division of Cancer Epidemiology and Genetics, National Cancer Institute, Bethesda, Maryland, United States of America; 10 Harvard University School of Public Health, Boston, Massachusetts, United States of America; Peking University First Hospital, CHINA

## Abstract

**Introduction:**

Gene-set analysis (GSA) is an approach using the results of single-marker genome-wide association studies when investigating pathways as a whole with respect to the genetic basis of a disease.

**Methods:**

We performed a meta-analysis of seven GSAs for lung cancer, applying the method META-GSA. Overall, the information taken from 11,365 cases and 22,505 controls from within the TRICL/ILCCO consortia was used to investigate a total of 234 pathways from the Kyoto Encyclopedia of Genes and Genomes (KEGG) database.

**Results:**

META-GSA reveals the systemic lupus erythematosus KEGG pathway *hsa05322*, driven by the gene region 6p21-22, as also implicated in lung cancer (p = 0.0306). This gene region is known to be associated with squamous cell lung carcinoma. The most important genes driving the significance of this pathway belong to the genomic areas *HIST1-H4L*, *-1BN*, *-2BN*, *-H2AK*, *-H4K* and *C2/C4A/C4B*. Within these areas, the markers most significantly associated with LC are rs13194781 (located within HIST12BN) and rs1270942 (located between *C2* and *C4A*).

**Conclusions:**

We have discovered a pathway currently marked as specific to systemic lupus erythematosus as being significantly implicated in lung cancer. The gene region 6p21-22 in this pathway appears to be more extensively associated with lung cancer than previously assumed. Given wide-stretched linkage disequilibrium to the area *APOM/BAG6/MSH5*, there is currently simply not enough information or evidence to conclude whether the potential pleiotropy of lung cancer and systemic lupus erythematosus is spurious, biological, or mediated. Further research into this pathway and gene region will be necessary.

## Introduction

Since the beginning of the 20^th^ century, lung cancer (LC) occurrence has been increasing rapidly and has become the most common cancer in males. It is the main cause of cancer-related death worldwide [1] and tobacco smoke is its major risk factor. The risk of developing LC in current smokers is 7.6 to 9.3 times higher compared to that of never smokers [2]. However, around every fourth LC case is not attributable to smoking [3]. A five-fold increased risk of developing early-onset LC in the presence of a family history of early-onset LC in any first-degree relatives has also been observed [4, 5]. This and other evidence has led to the general acceptance that a genetic component in early-onset LC development exists. However, an increased risk of developing LC has also been observed in patients with other disease, such as COPD, pneumonia, tuberculosis, or the autoimmune disorder *systemic lupus erythematosus* (SLE) [6, 7]. In the case of patients with SLE, an increased relative risk (RR) of developing LC was observed as being 1.68 (95%-CI: 1-33-2.13) [6]. In spite of multiform clinical manifestations and outcomes, it is generally accepted that genetics plays a role in SLE [8]. In light of the results of this investigation, we will discuss a shared genetic susceptibility as a possible connection between SLE and LC.

Genome-wide association studies (GWASs) have revealed that genomic variations at e.g. 5p15.33, 6p21-22 and 15q25 influence LC risk in European populations [9–16]. Further weakly associated single markers in at least 12 genes have been found given their known role within certain molecular mechanisms [17–21]. Since associated genes are elements of respective pathways, one may assume that nicotine dependency [14], inflammation [16, 22], or DNA repair [23], among others, play a role in an individual’s susceptibility to developing LC.

The usual approach to identify such molecular mechanisms with GWAS is primarily to investigate single-marker-association and then allocate these markers to genes and finally the genes to pathways. Doing so, either the marginal effect of a single marker and/or the sample size needs to be large, because a low genome-wide level of significance of 1 x 10^−7^ or smaller is needed owing to multiple testing. Gene-set analysis (GSA) strategies were proposed as complementary approaches in the investigation of the genetic basis of a disease using GWAS results [24–26], by seeking to identify sets of genes (GS) with sufficient enrichment of marker-specific significance for an association with a phenotype.

GSA approaches provide no effect estimates of the association, but only p-values (*p*_*GS*_). To pool the *p*_*GS*_-values of several GSAs, it is important to take into account the concordance across studies of all single-marker-association point estimates related to every gene in a considered gene set [27]. However, one only needs to correct for multiple testing using the lower number of GSs being investigated instead of the larger number of genotyped markers. Once a GS has been found to be significantly associated, a search may be conducted for the genes that drive its significance and for the hosted markers which are concordant across studies based on their observed associations.

Here we aimed to identify pathways taken from the Kyoto Encyclopedia of Genes and Genomes (KEGG) database [28] as being associated with LC. KEGG provides a collection of manually drawn pathway maps representing an up-to-date knowledge on the molecular interaction and reaction networks. This includes pathways for metabolisms (e.g. nicotinate and nicotinamide metabolism), for genetic information processing (e.g. DNA repair), for environmental information processing (e.g. Wnt signaling), for cellular processes (e.g. cell cycle), for organismal systems (e.g. circadian rhythm) and last but not least for human diseases (e.g. LC or SLE) [29]. We refrained from restricting the KEEG collection, because pathways that are potentially involved in the etiology of LC (examples are given above in brackets) are contained in every upper mentioned category.

Our subsequent goal was to determine the driving genes in the pathways identified in the first step. To this end, we combined the results of seven LC GWASs from the Transdisciplinary Research in Cancer of the Lung / International Lung Cancer Consortium (TRICL / ILCCO) in a meta-analysis.

## Materials and methods

### Description of studies

**Fig 1:**
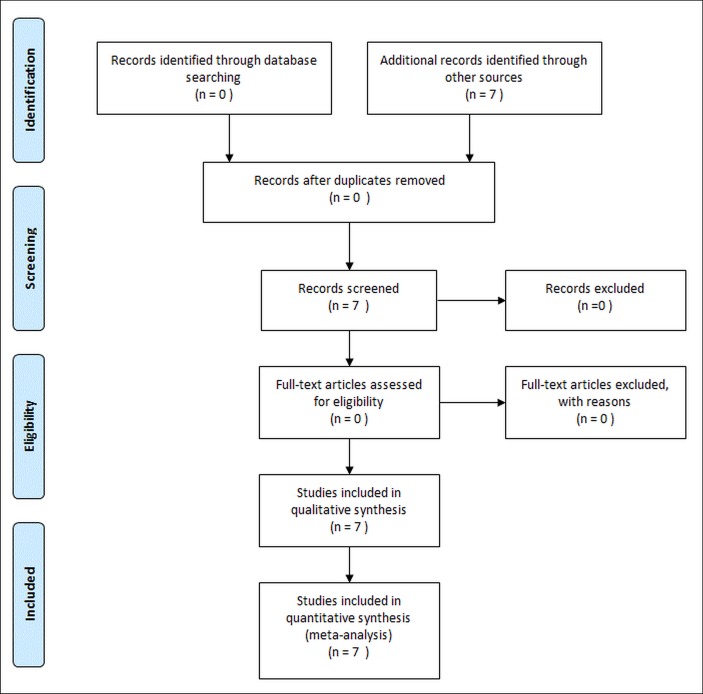
Study selection flow cart.

The meta-analysis was based on summary data from seven previously reported LC GWASs form TRICL / ILCCO (Fig 1). We included 11,365 LC cases and 22,505 controls of European descent in the analysis. An overview as well as study name abbreviations are given in Table 1. Details and references are provided Supplement S1 File.

**Table 1:** Characteristics of lung cancer GWASs of the International Lung Cancer Consortium (ILCCO).

Study	Cases	Controls	Location	Study design	Illumina genotyping platform	Number of SNPs
**Scanning phase**						
MDACC^a^	1 150	1 134	Texas, USA	Hospital-based case–control	317K	312 829
TORONTO^b^	331	499	Toronto, CA	Hospital-based case–control	317K	314 285
CE (IARC^c^)	1 854	2 453	Romania, Hungary, Slovakia, Poland, Russia, Czech Republic	Multicenter hospital-based case–control	317K, 370Duo	
GLC^d^	487	480	Germany	Population-based case–control (<50 years)	HumanHap550K	503 381
**Replication phase**						
DeCODE Genetics	830	11 228	Iceland	Population-based case–control	317K, 370Duo	290 386
HARVARD	984	970	Massachusetts, USA	Hospital-based case–control	610Quad	543 697
*NCI GWAS*						506 062
EAGLE^e^	1 920	1 979	Italy	Population-based case–control	HumanHap550v3_B, 610Quad	
ATBC^f^	1 732	1 271	Finland	Cohort	HumanHap550K, HumanHap610	
PLCO^g^	1 380	1 817	10 US Centers	Cohort-Cancer Prevention Trial	317K / 240S, HumanHap550v3_B, HumanHap610	
CPS-II^h^	697	674	all US states	Cohort	HumanHap550K, 610Quad	
**Overall**	**11 365**	**22 505**				

^a^ MD Anderson Cancer Center.

^b^ Toronto study by Lunenfeld-Tanenbaum Research Institute.

^c^ Central Europe Study of the International Agency for Research on Cancer.

^d^ German Lung Cancer Study.

^e^ Environment And Genetics in Lung cancer Etiology study.

^f^ Alpha-Tocopherol, Beta-Carotene Cancer Prevention study.

^g^ Prostate, Lung, Colon, Ovary screening trial.

^h^ Cancer Prevention Study II nutrition cohort.

### Strategy and methods

In the original GWASs, a log-additive mode of inheritance was fitted for each marker, adjusting for age, sex, smoking status, study center (if applicable), and the first three principal components to account for hidden genomic structure. The results of marker-by-marker association testing were used as input information for the GSAs.

For this meta-analysis, we set up a two-phase seamless design consisting of a screening phase and a replication phase. In the screening phase, the results of MDACC, TORONTO, GLC, and CE were combined, because GSA of these studies was performed for 234 KEGG pathways previously [30, 31]. In the replication phase, the results of the remaining studies NCI, deCODE, and HARVARD were combined to investigate only those pathways whose findings in the screening phase proved promising. If necessary, GSA was performed using the program ALIGATOR [32]. The method META-GSA [27] was performed to pool GSA results (p-values *p*_*GS*,*s*_) at each stage. The aim of META-GSA is to increase statistical evidence by pooling the *p-values p*_*GS*,*s*_ of GSAs, taking also into account the concordance of the signs of single-marker-association point estimates and related p-values of all markers (*p*_*m*,*s*_) assigned to genes contained in the GS [27]. The core element of this approach is a directed p-value (PDR), combining significance and direction of single markers and LD to other markers. Necessary estimates of LD were based on the genotype data of GLC, with imputation of missing markers based on the 1000-Genome Project [33], the 1000-GenomePilot 1-Panel or the HapMap3-Panel as available using the SNAP online tool [34].

The SNP-to-gene annotation (StG) for humans of the ENSEMBL database [35] was used. Markers with LD of at least r^2^≥0.8 to any marker inside a gene were additionally assigned to that gene [36]. All genes were then annotated to 234 gene sets from the KEGG database (gene-to-pathway annotation (GtP)).

Both phases can be considered as the first and the second stage of a seamless, adaptive study with interim selection of gene sets (“drop-loser design” [37]). The investigation of every KEGG pathway with a pooled *p*_*scr*._ < *β*_1_ = 1/234 in the screening phase was stopped early for futility. The significance, combining screening and replication phase, was assessed according to the “method based on the sum of p-values” (MSP) [37, 38]. The p-value was then calculated by the equation $p_{GS}=\beta_{1}(p_{scr.}+p_{rep.})-0.5\beta_{1}^{2}$. This *p*_*GS*_ needs to be corrected for multiple testing by taking into account the total number of 234 pathways. Due to pathway overlap we estimated the number of independent tests *t*_*eff*_ according to the lowest slope method (LSM) [39] considering all *p*_*scr*._-values of the screening phase. Applying a Bonferroni-like correction then yields the final p-value *p*_*GS*,*corr*._ = *min*(1,*t*_*eff*_ ⋅ *p*_*GS*_). Furthermore, META-GSA was also applied to all seven studies and all pathways surviving the screening phase to take into account the concordance of single-marker-association point estimates across all considered studies at the same time.

The next step was to identify the main genes driving the significance of gene sets (denoted as *p*_*GS*_–driving genes). Thus we contrasted the mean of PDRs across studies for each gene (${\bar{PDR}}_{g}$ as a measure of concordance) with pooled p-values regarding the gene-level statistics (*p*_*gene*_ as measure of significance, calculated according to Fisher’s χ^2^-method). To judge these findings adequately, we also calculated ${\bar{PDR}}_{g}$ for the known LC-related genes *CLTM1L*, *TERT*, *CHRNB4*, *CHRNA3*, *CHRNA5*, *MSH5*, *BAG6*, *RAD52* and *CDKN2B*. Within these genes we looked markers with a large mean of PDRs across studies (${\bar{PDR}}_{m}$).

Finally, we performed a sub-group meta-analysis for the one identified KEGG pathway according to histological subtype (AdenoLC, SqCLC, SCLC and LCLC), sex, age (older or younger than 50 years), and smoking behavior (current, former, ever and never smokers).

During this investigation the region 6p21-22 became of interest. Respective correlation of marker genotypes and gene expression (eQTL) was previously measured in non-neoplastic pulmonary parenchymal samples taken some distance from the primary tumor in LC patients [40]. We used the estimated correlation between every SNP located between 31.6MB and 32.2 MB (all within 6p21-22) and the expression of the genes *APOM*, *BAG6*, *MSH5* (reported as relevant in LC), *C2*, *C4B*, *SKIV2L*, *STK19* (closely located to genes driving the significance in this META-GSA application) and *TNXB* (reported as relevant for SLE), in total 5,572 estimated correlations. Estimating *t*_*eff*_ = 5309 independent tests (by LSM) yields a global threshold for significance of 1x10^-7^.

## Results

### Association of pathways: Screening and replication phase

Only three of the 234 pathways investigated revealed a p-value lower than the futility threshold and were selected for the replication phase: *hsa05322*: *systemic lupus erythematosus (SLE)*, *hsa00790*: *folate biosynthesis* and *hsa04940*: *type I diabetes mellitus* (Table 2). Only for the SLE pathway we were able to achieve a low p-value when combining screening and replication phase and correcting for multiple testing (*p*_*GS*,*corr*_ = 0.0615). Combining all seven studies in a single META-GSA, in order to take the concordance of single-marker-association point estimates of all studies into account adequately, yielded a p_GS_-value of 0.0306 for this SLE pathway. This indicates sufficient enrichment and satisfactory concordance of marker-specific significance for an association with LC.

**Table 2:** Significant results of META-GSA.

KEGG pathways	number of genes	screening		replication	MSP combination		all
		4 studies		3 studies			7 studies
	n_ genes_	*p*_*scr*._	*p*_*scr*.*corr*._^$^	*p*_*rep*._	*p*_*GS*_	*p*_*GS*,*corr*._^$^	*p*_*GS*_
*hsa05322* (SLE)	128	***0.0003	*0.0457	0.0857	***0.0004	0.0615	*0.0306
*hsa00790* (folate bio.)	13	***0.0003	0.0543	0.9122	***0.0046	0.6672	0.3154
*hsa04940* (T1DM)	42	***0.0011	0.1940	0.4890	***0.0024	0.3570	0.3952
231 other gene sets		>0.0043	futility	stopping			

*SLE*—systemic lupus erythematosus; *folate bio* folate biosynthesis; *T1DM*—type I diabetes mellitus, *MSP*—combined p-values according to the method based on the sum of p-values (adaptive designed approach for early futility stopping); *p*_*scr*._—p-value of the screening phase; *p*_*scr*.*corr*._—p-value of the screening phase corrected for multiple testing; *p*_*rep*._—p-value of the replication phase, *p*_*GS*_*—p-value of the gene set (combining p*_*scr*_ *and p*_*rep*_*); p*_*GS*,*corr*._*—p-value of the gene set* corrected for multiple testing; effective number of independent gene sets according the lowest slope method (LSM).

^$^: t_*eff*_ = 171.5.

* P ≤ 0.05.

** P ≤ 0.01.

*** P ≤ 0.001.

### Genes driving significance

Four genes of the SLE pathway (*HIST1-H4L*,*-1BN*, *-H2AK*, *-H4K*) and their close neighbor *HIST1H2BN* strike out by concordance of marker-specific association (${abs(\bar{PDR}}_{g})∼0.8$) across studies and a gene-level *p*_*gene –*_*value lower than* 0.01 (Table 3). All five genes belong to the histone cluster 1 and are closely located within 41 kb of each other on 6p22.1. Weaker concordance was observed for further two less significant genes (*p*_*gene -*_*value* < 0.05): C4A (${\bar{PDR}}_{g}$ = -0.41) and *C2* (${\bar{PDR}}_{g}$ = 0.33).

**Table 3:** Significance and concordance of selected genes of interest.

gene	location	number of studies with	concordance	significance
		*p*_*gene*,*study*_ < 5%	${\bar{PDR}}_{g}$	*p*_*gene*_
***significant genes belonging to the significant gene set hsa05322 (SLE)***				
***HIST1H4K***	6p22.1	2	-0.84	0.0056
***HIST1H2BN***	6p22.1	2	-0.80	0.0091
***HIST1H2AK***	6p22.1	2	-0.80	0.0091
***HIST1H1B***	6p22.1	2	+0.75	0.0093
***HIST1H2AL***	6p22.1	2	+0.75	0.0093
***C2***	6p21.3	2	+0.33	0.0109
***C4A***	6p21.3	1	-0.41	0.0319
***genes known to be associated with LC (for comparison only)***				
***CLPTM1L***	5q15.33	4	-0.53	< .0001
***TERT***	5q15.33	4	+0.49	0.0013
***CHRNB4***	15q24	3	-0.63	< .0001
***CHRNA3***	15q24	4	-0.58	< .0001
***CHRNA5***	15q24	3	-0.45	0.0009
***MSH5***	6p21.3	3	+0.67	< .0001
***BAG6***	6p21.3	--	+0.39	0.1425
***RAD52***	12p13.33	1	+0.23	0.3143
***CDKN2B***	9p21.3	--	-0.13	0.6729

*p*_*gene*,*study*_ is the study specific p-value for gene; ${\bar{PDR}}_{g}$ is the mean of study specific PDRs for a gene (95% random interval derived from all 16.000 assigned genes: [±0.306]); pooled *p*_*gene*_—*p*_*gene*,*study*_-values combined by Fisher’s inverse χ^2^-method.

### Markers driving significance

The markers rs13194781, rs1270942 and rs389884 are those with the largest ${\bar{PDR}}_{m}$-values (all >0.7) and the strongest associations with LC (in terms of OR). For rs13194781, which is located within *HIST1H2BN* (ENSEMBL definition), an OR of 1.23 (p = 0.0032) was estimated. The markers rs1270942 and rs389884 are perfect proxies for each other according to the 1000-Genome Pilot 1-panel [33]. They are closely located upstream of C2 and downstream of C4A, respectively. There is no LD with the first marker rs13194781 (Table 4).

**Table 4:** Markers with <0.5 in genes of interest on 6p21-22.

SNP	allocated to	Position	MAF	r^2^ to		D‘ to		${\bar{PDR}}_{m}$	LC		SqCLC	
				(A)	(B)	(A)	(B)		OR	p-value	OR	p-value
rs200991	*HIST1++*	27847716	0.12^a^	0.646^b^		1^a^		0.598	1.14	0.0021	1.16	3.1×10^−5^
**rs13194781 (A)**	*HIST1++*	27847861	0,08	1		1		0.719	1.23	0.0032	1.22	9.7×10^−6^
rs9262143	*MDC1*	30685004	0.16^§^					0.769	1.25	0.0027	1.25	1.3×10^−7^
rs3094127	*MDC1*	30729670	0.18					0.664	0.84	0.0029	1.10	4.0×10^−2^
rs3128982	*HCP5*	31449414	0.30					0.578	1.07	0.0032	1.12	1.1×10^−3^
rs3117582	*BAG6*	31652743	0.09^b^		0.881^b^		1^b^	0.485	1.27	0.0049	1.30	4.5×10^−10^
rs3131379	*MSH5*	31753256	0.09^b^		0.881^b^		1^b^	0.461	1.20	0.0074	1.28	3.8×10^−7^
rs652888	*C2*	31883457	0.17		0.336^b^		1^b^	0.538	1.14	0.0013	1.18	1.3×10^−4^
rs535586	*C2*	31892560	0.35		0.131^b^		1^b^	0.606	1.09	0.0001	1.11	1.2×10^−3^
rs659445	*C2*	31896527	0.35		0.131^b^		1^b^	0.711	1.09	3.7×10^−6^	1.10	3.1×10^−3^
**rs1270942 (B)**	*C2*	31951083	0.09^b^		1		1^b^	0.728	1.27	0.0090	1.29	5.8×10^−6^
rs438999	*C2*	31960529	0.06		0.005^b^		1^b^	-.517	0.91	0.0027	0.85	1.0×10^−2^
rs454212	*C4A*	31966595	0.08					-.556	0.95	0.0034	0.84	1.7×10^−2^
**rs389884**	*C4A*	31973120	0.09^b^		1^$^		1^b^	0.724	1.27	0.0080	1.28	7.2×10^−6^

Odds ratios (OR), corresponding p-values from a random effects meta-analysis model; single study ORs were adjusted for age, sex, smoking and genetic background; r^2^ and D’ were calculated according to the HapMap3-panel.

(^a^) or the 1000 Genome Pilot 1-panel.

(^b^) using SNAP Version 2.2; HIST1++ denotes the gene cluster HIST1-H4L/H2BN/H2AK/H2BN/H4K; LC—lung cancer (all histological subtypes), SqCLC – squamous-cell lung cancer; markers with largest ${\bar{PDR}}_{m}$ with genes driving the significance of the SLE gene set (HIST1++, C2 and C4A) are printed in bold. Position of SNPs is given according to NCBI Build 37. MAF … minor allele frequencies in controlls.

### Subgroup meta-analysis

We revealed more evidence for an association of the SLE pathway with AdenoLC (*p*_*GS*_ = 0.0030) than for any other histotype. We also found the association to be significant in women (*p*_*GS*_ = 0.0112) but not in men (*p*_*GS*_ = 0.1453) and in older cases (*p*_*GS*_ = 0.0002) but not in younger (*p*_*GS*_ = 0.0588). No significant association was observed when stratifying according to smoking behavior (Table 5). Significance within the considered subgroups is driven by same *p*_*GS*_-driving genes of the region 6p22.1–22.2 as in the total sample (C2 and the genes of the histone 1 cluster). Also, most of the more moderate concordant genes that drive significance of *hsa05322* in at least one of the considered subgroups are histone-coding genes.

**Table 5:** Subgroup analysis for *hsa05322*: histological subtypes, sex, age, smoking.

*hsa05322*: SLE	META-GSA	Gene	Location	concordance	significance
	*p*_*GS*_			${\bar{PDR}}_{g}$	*p*_*gene*_
AdenoLC	**0.0030**	HIST2-1q21.2	1q21.2	-0.6	0.1666
SqCLC	**0.0376**	H2AFV	7p13	0.5	0.7209
SCLC	0.0626	C1QA	1p36.12	-0.5	0.7101
		HIST2-1q21.2	1q21.2	-0.5	0.0577
		ELANE	19p13.3	0.5	0.4864
		HIST1-6p22.2	6p22.2	0.5	0.2177
		HIST1-6p22.2	6p22.2	0.5	0.2177
LCLC	0.2056	--			
male	0.1453	HIST1H3C	6p22.2	-0.5	0.3726
female	**0.0112**	HIST1H2AL	6p22.1	0.5	0.1229
old (>50)	**0.0002**	HIST1-6p22.1a	6p22.1	-0.7	0.0054
		HIST1-6p22.1b	6p22.1	0.5	0.1578
		C2	6p21.3	0.5	0.0013
		H2AFV	7p1	0.6	0.4005
young (≤50)	0.0588	--			
current smokers	0.3563	HIST1-6p22.1a	6p22.1	0.4	0.1720
		H3F3C	12p11.21	0.4	0.5821
		HIST3H3	1q42	0.4	0.6468
		HIST1-6p22.1b	6p22.1	0.4	0.2028
		HIST1-6p22.1c	6p22.1	0.4	0.3375
former smokers	0.4691	--			
ever smokers	0.5132	HIST1-6p22.1a	6p22.1	0.5	0.0462
		HIST1-6p22.1c	6p22.1	0.5	0.1587
never smokers	0.5429	FCGR3A	1q23	-0.5	0.2300
		CTSG	14q11.2	-0.5	0.3403

Listed are genes, respectively regions containing genes with $abs({\bar{PDR}}_{g})\geq 0.5$.

HIST2-1q21.2: HIST2H2AA3 / HIST2H2AA4 / HIST2H3C / HIST2H4B.

HIST3-1q42: HIST3H2A / HIST3H2BB / HIST3H3.

HIST1-6p22.1a: HIST1H4K / HIST1H2AK / HIST1H2AL / HIST1H2BM / HIST1H2BN / HIST1H3I / HIST1H4L / HIST1H3J / HIST1H4J (27.800K).

HIST1-6p22.1b: HIST1H2AG / HIST1H2BK (27.150 K).

HIST1-6p22.1c: HIST1H2BI / HIST1H3G / HIST1H4H (26.280 K).

HIST1-6p22.2: HIST1H3E / HIST1H2AE / HIST1H2BG / HIST1H4E (26.200 K).

The numbers in brackets are the approximate locations according to dbGENE.

### SNP ⨯ eQTL correlation

**Fig 2:**
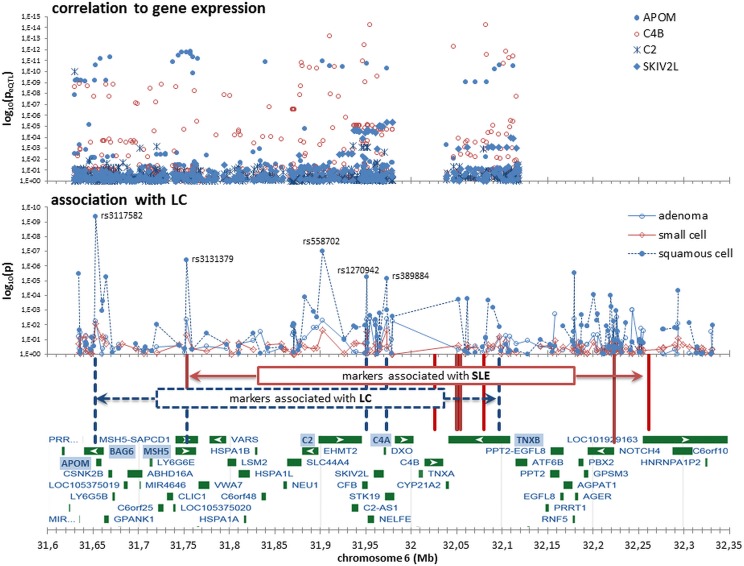
Association and correlation with gene expression in the chromosome 6p21-22 region. *LC*—lung cancer, *SLE*—systemic lupus erythematosus; correlation to gene expression: pooled p-values as reported by Nguyen et al., 2014 [40]; association with LC: pooled p-values as reported by Timofeeva et al. 2012 [13].

Both aforementioned SNPs belonging to *C2/C4A*, rs1270942 and rs389884, are significant correlated with the expression of the gene *APOM* (p<10^−13^), which is located about 500 kb away (Fig 2). However, the expression pattern is this region is puzzling, since other markers within *C2* (rs537160, rs622871, rs630379) are also correlated with the gene expression in non-neoplastic samples of LC patients of the neighboring gene *C4B* (not part of the investigated KEGG pathway, although related to SLE). It is also remarkably that the correlation of SNPs belonging to *C2/C4A* with the expression of *C2* is less significant (p ~10^−3^) than with the expression of SKIV2L (p ~10^−5^), which is not related to SLE.

## Discussion

We could demonstrate an accumulation of genomic association with LC in the KEGG pathway *hsa05322*, which comprises genes related to SLE. This suggests some cross-phenotype (CP) association with LC and SLE. The significance was higher in the subgroup of AdenoLC patients than within other histological subtypes and in women compared to men. This fits our expectations in view of women, who predominantly develop AdenoLC, are more often affected with SLE than men [41], who predominantly develop smoking-related SqCLC [1, 42].

All *p*_*GS*_–driving genes identified in this meta-analysis are located within or next to the major histocompatibility complex (MHC) on chromosome 6p21-22 (Fig 2), albeit in two separate areas, about 3000 kb apart. The first area comprises the genes of histone cluster I: *HIST1-H4L*, *-1BN*, *-2BN*, *-H2AK*, *-H4K* (the strongest associated marker is rs13194781; OR = 1.23, p = 0.0032). It is well known that a variety of histone related modifications are either related to cancer or to SLE, or to both [8, 43]. They play a role e.g. in DNA repair, cell cycle or gene expression [8, 44], which by themselves are associated to LC or SLE, respectively [23, 45]. Interestingly enough, we detected associations to LC of the DNA signature of histone coding genes, rather than with respect to some kind of epigenetic outcome.

The second area comprises the genes *C2*, *C4A*, and *C4B* (the strongest associated markers are rs1270942 and rs389884; OR = 1.27, p = 0.009). It is well established, that reduced gene expression of C2 and C4A can predispose to SLE [46]. This two genes, and perhaps also *C4B*, are involved in the clearance of apoptotic bodies [8]. This is in turn crucially important for controlling inflammation, which plays a role in the development of LC [3].

However, the identification of disease-relevant genes in the MHC region (6p21–6p22) and far beyond is complicated owing to the strong and extensive LD across both common and rare haplotypes [47]. Hence any observed CP association will probably tag plenty of genes. An association of the gene area APOM/*BAG6/MSH5* in the MHC region with LC has previously been reported, which is strongest for SqCLC and AdenoLC [9, 13]. The strongest associations with SqCLC in this area was previously reported for the markers rs3117582 (located within *BAG6 and APOM;* OR = 1.3, p = 4.5×10^−10^), which was found associated also with SLE (OR = 2.2, p = 4.2×10^-21^) [48]. This marker is about 220 kB apart but in strong LD with the newly identified markers rs1270942 and rs389884 (located close to C2; Table 4 and Fig 2). More important, a highly significant correlation between markers of the area *C2/C4A/C4B* with the expression of the gene *APOM* in non-neoplastic samples taken from LC patients was also recently reported [40] (Fig 2). APOM is involved in lipid transport and is linked with high-density lipoprotein cholesterol in the pathogenesis of emphysema, which is on the other hand considered as associated with LC [49, 50]. But other explanations of the observed associations have been given, too; for instant a connection to embryonic lethality with defects in the development of the lung (related to the function of *BAG6*) or deficits in mismatch excision repair (related to the function of *MSH5*) [13]. Moreover, the association of MSH5 with SLE was reported as not shared with other autoimmune/inflammatory diseases [51].

Apart from all this, some remarks about the applied method need to be made. The whole approach is an intensive investigation of p-values, which—in the context of this project—are indicators of evidence for or against the rejection of a null-hypothesis of no genetic association. We used the program ALIGATOR to perform GSA, which circumvents bias due to uneven counts of markers per gene as well as genes per gene set [32]. Choosing another algorithm would probably lead to different results [31]. In addition, a p-value can be used to justify the existences of an association; however it is not solely determined by the strength of the observed effect, but also by factors like sample size, the used statistical model and the applied test procedure. Hence we can present significance of our findings but are unable to estimate the part of LC risk that can be attributed to the identified genes or gene sets.

## Conclusion

We were able to identify CP risk factors by first pooling results of gene set analyses and looking afterwards for those genes driving the significance of discovered gene sets. In doing so, we have discovered a pathway that is currently marked as specific to SLE as being significantly implicated in LC. The gene region 6p21-22 in this pathway appears to be more extensively associated with lung cancer than previously assumed. Given wide-stretched linkage disequilibrium to the area *APOM/BAG6/MSH5*, there is currently simply not enough information or evidence to conclude whether the potential pleiotropy of LC and SLE is spurious, biological, or mediated. Further research into this pathway and gene region will be necessary.

## Supporting information

S1 File Detailed study description. (DOCX)

S2 File PRISMA Checklist. (DOCX)

S3 File Meta-analysis on Genetic Association Studies Checklist | PLOS ONE. (DOCX)
