# Supplementary material for: Gene-set meta-analysis of lung cancer identifies pathway related to systemic lupus erythematosus
Source: PLoS One. 2017 Mar 8;12(3):e0173339. doi: 10.1371/journal.pone.0173339 (PMC5342225; doi:10.1371/journal.pone.0173339)
Supplement: S1 File — (DOCX) [file pone.0173339.s001.docx]

**Shared genetic association of lung cancer and systemic lupus
erythematosus discovered in a gene set meta-analysis**

Albert Rosenberger et al. **Supplementary Material**

# Study description

# Studies under NCI umbrella

Detailed characteristic of NCI studies is presented elsewhere([1](#_ENREF_1)). Briefly it includes samples from studies:

1. The Environment and Genetics in Lung cancer Etiology (EAGLE) is a population-based case-control study including 2,100 lung cancer cases and 2,120 healthy controls enrolled in Italy between 2002 and 2005 ([2](#_ENREF_2)).
2. The Alpha-Tocopherol, Beta-Carotene Cancer Prevention Study (ATBC) is a randomized primary prevention trial including 29,133 male smokers enrolled in Finland between 1985 and 1993([3-5](#_ENREF_3)).
3. The Prostate, Lung, Colon, Ovary Screening Trial (PLCO) is a randomized trial including 150,000 individuals enrolled in ten U.S. study centers between 1992 and 2001([6](#_ENREF_6)).
4. The Cancer Prevention Study II Nutrition Cohort (CPS-II) is a cohort study including over 183,000 subjects enrolled by the American Cancer Society between 1992 and 2001 across all U.S. states ([5](#_ENREF_5), [7](#_ENREF_7)).

The lung cancer diagnosis in the NCI GWAS was based on clinical criteria and confirmed by pathology reports from surgery, biopsy or cytology samples in approximately 95% of cases and on clinical history and imaging for the remaining 5%. Tumor histology was coded according to the International Classification of Disease fir Oncology (ICD-O). Overall, between 10% and 50% of all diagnosis from the NCI GWAS were centrally reviewed by expert lung pathologies from NCI. For the histology analysis the following codes were used - non small cell carcinoma: adenocarcinoma (8140, 8251, 8255, 8260, 8310, 8323, 8480, 8481, and 8570), bronchioloalveolar carcinoma (8250, 8252, and 8253), large cell carcinoma (8012 and 8031), squamous cell carcinoma (8052, 8070, 8071, 8072, 8073, and 8074), and other non-small cell carcinoma (ONSCLC) (8010, 8020, 8022, 8032, 8033, 8046, 8050, 8490, 8550, and 8560); small cell carcinoma: (NOS 804 8041/3): 8042/3 oat cell carcinoma, 8043/3 small cell carcinoma, fusiform cell, 8044/3 Small cell carcinoma, intermediate cell, 8045/3, combined small cell carcinoma.

# Central Europe study

The Central Europe Study is a multicenter hospital-based case-control study ([8-10](#_ENREF_8)). The diagnosis of lung cancer for all studies was established based on clinical criteria and confirmed by pathology reports from surgery, biopsy or cytology samples. The International Classification of Diseases for Oncology (ICD-O) was used. No information on histology was available for the case-control lung cancer study from Estonia. Therefore Estonia study did not contribute to the histology subgroup analysis.

# Toronto study

The Toronto study was conducted in the Great Toronto Area between 1997 and 2002 ([11](#_ENREF_11)). Cases were recruited at the hospitals in the network of University of Toronto and Lunenfeld-Tanenberg Research Institute (LTRI). At the time of recruitment in the clinical setting, provisional diagnoses of lung carcinoma were first assigned based on clinical criteria. Diagnoses for all cases included were histologically confirmed by the reference pathologist who is a specialist in pulmonary pathology, based on review of pathology reports from surgery, biopsy or cytology samples in 100% of cases. Diagnostic classification was done initially according to ICD-9, ICD-10, and ICD for oncology-2, and subsequently converted to ICD-O-3. Tumors were grouped into the major categories included in this analysis according to primary cancer type based on the ICD-3 definitions. Controls were randomly selected from individual visiting family medicine clinics and Ministry of Finance Municipal Tax Tapes. All subjects were interviewed using a standard questionnaire and information on lifestyle risk factors, occupational history; medical and family history was collected. Blood samples were collected from more than 85% of the subjects.

# deCODE Genetics

The Icelandic lung cancer study population has been described previously ([12](#_ENREF_12), [13](#_ENREF_13)). Briefly, according to the population-based Icelandic Cancer Registry (www.krabbameinsskra.is), a total of 4,252 lung cancer patients were diagnosed from January 1, 1955, to December 31, 2010. The registry receives information from all pathology and cytology laboratories in Iceland, in addition to hematology laboratories, hospital wards, private medical practitioners and other individual health care workers. Approximately 94.5% of diagnoses in the ICR have histological confirmation. Histological subtype is registered in the Icelandic Cancer Registry using the ICD- 03 classification. For the histology analysis the following codes were used - adenocarcinoma: 8140/3, 8250/3, 8260/3, 8310/3, 8480/3 8560/3; large cell carcinoma: 8012/3, 8031/3; squamous cell carcinoma: 8070/3, 8071/3, 8072/3, 8074/3; other non-small cell carcinomas: 8010/3, 8020/3, 8021/3, 8032/3, 8230/3; small cell carcinoma: 8041/3, 8042/3, 8043/3, 8044/3, 8045/3. Recruitment of both prevalent and incident cases was initiated in the year 1998, the recruitment is ongoing and DNA samples from lung cancer cases are subjected to whole-genome genotyping as they are collected.

The controls used in this study consisted of individuals from other ongoing genome-wide association studies at deCODE, age and sex matched to the group of cases. No individual disease group accounts for more than 10% of the total control group.

Quality control for chip genotyping: SNPs were excluded if they had (i) yield lower than 95%, (ii) minor allele frequency less than 1% in the population or (iii) significant deviation from Hardy-Weinberg equilibrium in the controls (*P* < 0.001), (iv) if they produced an excessive inheritance error rate (*i.e.* >0.001), (v) if there was substantial difference in allele frequency between chip types (from just a single chip if that resolved all differences, but from all chips otherwise). All samples having a call rate <97% were excluded from the analysis.

# Harvard Lung Cancer Study

For the Harvard Lung Cancer Susceptibility Study, details of participant recruitment have been described previously ([14](#_ENREF_14), [15](#_ENREF_15)). The genotyped dataset includes 1,000 cases and 1,000 controls. Cases were patients over the age of 18 years, with newly diagnosed and histologically confirmed primary non-small cell lung cancer; controls were healthy non-blood-related family members and friends of patients with cancer or with cardiothoracic conditions undergoing surgery. The histological classification was done by two staff pulmonary pathologists at the Massachusetts General Hospital according to the International Classification of Diseases for Oncology (ICDO3). For the histology analysis the following codes were used - adenocarcinoma: 8140/3, 8250/3, 8260/3, 8310/3, 8480/3 8560/3; large cell carcinoma: 8012/3, 8031/3; squamous cell carcinoma: 8070/3, 8071/3, 8072/3, 8074/3; other non-small cell carcinomas: 8010/3, 8020/3, 8021/3, 8032/3, 8230/3; and small cell carcinoma: 8041/3, 8042/3, 8043/3, 8044/3, 8045/3. A general quality control procedure for GWAS data was performed on the genotyped dataset. The procedure included assessing the failure rate per individual and per SNP, identifying individuals with discordant gender information, assessing the degree of the relatedness and identifying population outliers.

# MDACC

Cases and controls were ascertained from a case-control study that has been ongoing at the U.T. M.D. Anderson Cancer Center since 1997. Cases are newly diagnosed, histologically-confirmed patients presenting at M.D. Anderson Cancer and who had not previously received treatment other than surgery. Histological information about tumors was derived by chart review from medical records. Tumors were histologically classified as adenocarcinoma, squamous cell carcinoma, large cell carcinoma, mixed histologies, or unclassified NSCLC([16](#_ENREF_16), [17](#_ENREF_17)). Small cell carcinomas were excluded from the genome-wide association analysis conducted at M.D. Anderson Cancer Center. Controls are healthy individuals seen for routine care at Kelsey- Seybold Clinics; the largest physician group-practice plan in the Houston Metropolitan area. Controls were frequency matched to cases according to their smoking behavior, age in 5 year categories, ethnicity, and sex. Former smoking controls were further frequency matched to former smoking cases according to the number of years since smoking cessation (in 5 year categories).

# German Lung Cancer study (GLC)

The German Lung Cancer GWA study was made up of three independent German studies as detailed below: in total 506 incident lung cancer cases (LUCY-study: n=305, Heidelberg lung cancer case-control study: n=201) were compared to 480 population controls (KORA surveys KORA). After excluding individuals with missing values and potentially related individuals, 487 cases and 480 controls entered the data analysis for the TRICL meta-analysis project.

LUCY (LUng Cancer in the Young) is a multicenter study with 31 recruiting hospitals in Germany ([18](#_ENREF_18), [19](#_ENREF_19)). The study is conducted by the Institute of Epidemiology, Helmholtz Zentrum Muenchen, and the Department of Genetic Epidemiology, Medical School, University of Göttingen). The LUCY-study provides access to a nationwide, population based family and a case-control sample (control population KORA, described below) of lung cancer patients aged 50 years or younger at diagnosis. Detailed epidemiologic data have been collected including data on medical history, education, family history of cancer and smoking exposure by phase assessment. Blood samples are taken and DNA and lymphoblastoid cell lines are prepared of all cases and controls and of parts of the relatives. Phenotype data of 847 young patients with primary lung cancer and 5524 relatives have been collected. All cases were histologically confirmed and classified by a pathologist as adenocarcinoma, squamous cell carcinoma, large cell carcinoma, small cell carcinoma, other or unclassified histology.

Heidelberg lung cancer case-control study is an ongoing hospital based case-control study ([19](#_ENREF_19), [20](#_ENREF_20)). The German Cancer Research Center (DKFZ) has recruited over 2000 lung cancer cases at and in collaboration with the Thoraxklinik Heidelberg, including 300 lung cancer cases with onset of disease at the age of ≤ 50. The histological classification was done by a pathologist, according to the WHO International Histological Classification of Tumours (ICD-O) current at the time of recruitment. Approximately 750 hospital-based controls have also been recruited. Data on occupational exposure, tobacco smoking, educational status, and for a subgroup also on family history of lung cancer, assessed by a self-administered questionnaire is available. Blood samples have been taken, and DNA has been extracted.

KORA (Cooperative health research in the Region of Augsburg) survey is a population-based KORA platform established by the Helmholtz Center Munich ([21](#_ENREF_21)). In total, four population based health surveys have been conducted during 1984/85-1999/2001. Overall 18000 participants in the age range between of 25 and 74 years at first interview were recruited. Detailed information on demographic characteristics, medical history, history of tobacco consumption and lifetime occupation together with biological materials were collected for more than 16000 probands.

# Genotyping, Phenotyping, Quality control

In each of these studies, SNP genotyping had been performed using Illumina HumanHap 317K BeadChips, HumanHap550K or 610Quad arrays. Further details about selection criteria, cancer diagnosis, genotyping and quality control in each study are provided as supplementary material. Lung cancer diagnosis in most studies was based on histopathology or cytology but for a minority of study participants on clinical history and imaging ([22](#_ENREF_22)).

Samples were excluded if (i) the average call rate was < 90%, (ii) there was sex discordance (threshold of heterozygosity > 10% for males and < 20% for females), (iii) unexpected duplicates and evidence of first-degree relative relationships from identity-by-descent values, (iv) heterozygosity rates for autosomal chromosomes exceeded 6 standard deviation of the mean, (v) based on STRUCTURE analysis ([23](#_ENREF_23)) subjects were less than 80% European ancestry and (vi) detected as outliers based on principal component analysis (PCA) using EIGENSTRAT ([24](#_ENREF_24)). SNPs were excluded if (i) genotyped call rate was less than 95%. Test for Hardy-Weinberg equilibrium (HWE) were also performed, but no SNPs were excluded based on this test. However, each study center was requested to report this information together with results of the analysis for the specific study.

# Financial Support:

This study was supported by a grant from the National Institute of Health (NIH) (U19CA148127). The Toronto study was supported by Canadian Cancer Society Research Institute (020214), Ontario Institute of Cancer and ILCCO data management was supported by the Cancer Care Ontario Chair Award to R.H. The German Lung Cancer Study (GLC) consists of three data sets. The Heidelberg Lung Cancer Study was in part supported by a grant (70-2919) from the Deutsche Krebshilfe. The KORA Surveys were financed by the Helmholtz-Gemeinschaft (HGF) Munich. The LUng Cancer in the Young (LUCY) study was funded in part by the National Genome Research Network (NGFN), the Deutsche Forschungsgemein­schaft DFG (BI 576/2-1; BI 576/2-2), the HGF and the Federal Office for Radiation Protection (BfS: STSch4454). Genotyping was performed in the Genome-Analysis-Center (GAC) of the Helmholtz Zentrum München (HMGU). Support for the Central Europe, HUNT2/Tromsø and CARET genome-wide studies was provided by Institut National du Cancer, France. Support for the HUNT2/Tromsø genome-wide study was also provided by the European Community (Integrated Project DNA repair, LSHG-CT- 2005-512113), the Norwegian Cancer Association and the Functional Genomics Programme of Research Council of Norway. Support for the Central Europe study, Czech Republic, was also provided by the European Regional Development Fund and the State Budget of the Czech Republic (RECAMO, CZ.1.05/2.1.00/03.0101). The lung cancer GWAS from Estonia was partly supported by a FP7 grant (REGPOT 245536), by the Estonian Government (SF0180142s08), by EU RDF in the frame of Centre of Excellence in Genomics and Estoinian Research Infrastructure’s Roadmap and by University of Tartu (SP1GVARENG). The Environment and Genetics in Lung Cancer Etiology (EAGLE), the Alpha- Tocopherol, Beta-Carotene Cancer Prevention Study (ATBC) and the Prostate, Lung, Colon, Ovary Screening Trial (PLCO) studies and the genotyping of ATBC, the Cancer Prevention Study II Nutrition Cohort (CPS-II) and part of PLCO were supported by the Intramural Research Program of National Institute of Health (NIH), National Cancer Institute (NCI), Division of Cancer Epidemiology and Genetics. ATBC was also supported by U.S. Public Health Service contracts (N01-CN-45165, N01-RC-45035 and N01-RC-37004) from the NCI. PLCO was also supported by individual contracts from the NCI to the University of Colorado Denver (NO1-CN-25514), Georgetown University (NO1-CN-25522), Pacific Health Research Institute (NO1-CN-25515), Henry Ford Health System (NO1-CN-25512), University of Minnesota (NO1-CN-25513), Washington University (NO1-CN-25516), University of Pittsburgh (NO1- CN-25511), University of Utah (NO1-CN-25524), Marshfield Clinic Research Foundation (NO1-CN-25518), University of Alabama at Birmingham (NO1-CN-75022, Westat, Inc. NO1-CN-25476), University of California, Los Angeles (NO1-CN-25404). The Cancer Prevention Study II Nutrition Cohort was supported by the American Cancer Society. The NIH Genes, Environment and Health Initiative (GEI) partly funded DNA extraction and statistical analyses (HG-06- 033-NCI-01 and RO1HL091172-01), genotyping at the Johns Hopkins University Center for Inherited Disease Research (U01HG004438 and NIH HHSN268200782096C) and study coordination at the GENEVA Coordination Center (U01 HG004446) for EAGLE and part of PLCO studies. Funding for the MD Anderson Cancer Study was provided by NIH grants (P50 CA70907, R01CA121197, RO1 CA127219, U19 CA148127, RO1 CA55769) and CPRIT grant (RP100443). Genotyping services were provided by the Center for Inherited Disease Research (CIDR). CIDR is funded through a federal contract from the NIH to The Johns Hopkins University (HHSN268200782096C). The Harvard Lung Cancer Study was funded by Funded by NHI (CA074386, CA092824, CA090578).

# References

1. Gazdar AF, Shigematsu H, Herz J, Minna JD. Mutations and addiction to EGFR: the Achilles 'heal' of lung cancers? Trends in molecular medicine. 2004;10:481-6.

2. Landi MT, Consonni D, Rotunno M, Bergen AW, Goldstein AM, Lubin JH, et al. Environment And Genetics in Lung cancer Etiology (EAGLE) study: an integrative population-based case-control study of lung cancer. BMC public health. 2008;8:203.

3. Kataja-Tuomola MK, Kontto JP, Mannisto S, Albanes D, Virtamo JR. Effect of alpha-tocopherol and beta-carotene supplementation on macrovascular complications and total mortality from diabetes: results of the ATBC Study. Annals of medicine. 2010;42:178-86.

4. Aad G, Abbott B, Abdallah J, Abdel Khalek S, Abdinov O, Aben R, et al. Measurements of four-lepton production at the Z resonance in pp collisions at sqrt[s] = 7 and 8 TeV with ATLAS. Physical review letters. 2014;112:231806.

5. Landi MT, Chatterjee N, Yu K, Goldin LR, Goldstein AM, Rotunno M, et al. A genome-wide association study of lung cancer identifies a region of chromosome 5p15 associated with risk for adenocarcinoma. Am J Hum Genet. 2009;85:679-91.

6. Hayes RB, Sigurdson A, Moore L, Peters U, Huang WY, Pinsky P, et al. Methods for etiologic and early marker investigations in the PLCO trial. Mutation research. 2005;592:147-54.

7. Calle EE, Rodriguez C, Jacobs EJ, Almon ML, Chao A, McCullough ML, et al. The American Cancer Society Cancer Prevention Study II Nutrition Cohort: rationale, study design, and baseline characteristics. Cancer. 2002;94:500-11.

8. McKay JD, Hung RJ, Gaborieau V, Boffetta P, Chabrier A, Byrnes G, et al. Lung cancer susceptibility locus at 5p15.33. NatGenet. 2008;40:1404-6.

9. Wang Y, Broderick P, Webb E, Wu X, Vijayakrishnan J, Matakidou A, et al. Common 5p15.33 and 6p21.33 variants influence lung cancer risk. NatGenet. 2008;40:1407-9.

10. Brennan P, Crispo A, Zaridze D, Szeszenia-Dabrowska N, Rudnai P, Lissowska J, et al. High cumulative risk of lung cancer death among smokers and nonsmokers in Central and Eastern Europe. Am J Epidemiol. 2006;164:1233-41.

11. Hung RJ, McKay JD, Gaborieau V, Boffetta P, Hashibe M, Zaridze D, et al. A susceptibility locus for lung cancer maps to nicotinic acetylcholine receptor subunit genes on 15q25. Nature. 2008;452:633-7.

12. Rafnar T, Sulem P, Besenbacher S, Gudbjartsson DF, Zanon C, Gudmundsson J, et al. Genome-wide significant association between a sequence variant at 15q15.2 and lung cancer risk. Cancer Res. 2011;71:1356-61.

13. Thorgeirsson TE, Geller F, Sulem P, Rafnar T, Wiste A, Magnusson KP, et al. A variant associated with nicotine dependence, lung cancer and peripheral arterial disease. Nature. 2008;452:638-42.

14. Asomaning K, Miller DP, Liu G, Wain JC, Lynch TJ, Su L, et al. Second hand smoke, age of exposure and lung cancer risk. Lung Cancer. 2008;61:13-20.

15. Su L, Zhou W, Asomaning K, Lin X, Wain JC, Lynch TJ, et al. Genotypes and haplotypes of matrix metalloproteinase 1, 3 and 12 genes and the risk of lung cancer. Carcinogenesis. 2006;27:1024-9.

16. Bosken CH, Wei Q, Amos CI, Spitz MR. An analysis of DNA repair as a determinant of survival in patients with non-small-cell lung cancer. J Natl Cancer Inst. 2002;94:1091-9.

17. Amos CI, Wu X, Broderick P, Gorlov IP, Gu J, Eisen T, et al. Genome-wide association scan of tag SNPs identifies a susceptibility locus for lung cancer at 15q25.1. Nat Genet. 2008;40:616-22.

18. Rosenberger A, Illig T, Korb K, Klopp N, Zietemann V, Wolke G, et al. Do genetic factors protect for early onset lung cancer? A case control study before the age of 50 years. BMC Cancer. 2008;8:60.

19. Sauter W, Rosenberger A, Beckmann L, Kropp S, Mittelstrass K, Timofeeva M, et al. Matrix metalloproteinase 1 (MMP1) is associated with early-onset lung cancer. Cancer epidemiology, biomarkers & prevention : a publication of the American Association for Cancer Research, cosponsored by the American Society of Preventive Oncology. 2008;17:1127-35.

20. Dally H, Gassner K, Jager B, Schmezer P, Spiegelhalder B, Edler L, et al. Myeloperoxidase (MPO) genotype and lung cancer histologic types: the MPO -463 A allele is associated with reduced risk for small cell lung cancer in smokers. International journal of cancer Journal international du cancer. 2002;102:530-5.

21. Wichmann HE, Gieger C, Illig T. KORA-gen--resource for population genetics, controls and a broad spectrum of disease phenotypes. Gesundheitswesen. 2005;67 Suppl 1:S26-S30.

22. Timofeeva MN, Hung RJ, Rafnar T, Christiani DC, Field JK, Bickeboller H, et al. Influence of common genetic variation on lung cancer risk: meta-analysis of 14 900 cases and 29 485 controls. Human molecular genetics. 2012;21:4980-95.

23. Falush D, Stephens M, Pritchard JK. Inference of population structure using multilocus genotype data: linked loci and correlated allele frequencies. Genetics. 2003;164:1567-87.

24. Price AL, Patterson NJ, Plenge RM, Weinblatt ME, Shadick NA, Reich D. Principal components analysis corrects for stratification in genome-wide association studies. Nat Genet. 2006;38:904-9.
