# Supplementary material for: Gene-set meta-analysis of lung cancer identifies pathway related to systemic lupus erythematosus
Source: PLoS One. 2017 Mar 8;12(3):e0173339. doi: 10.1371/journal.pone.0173339 (PMC5342225; doi:10.1371/journal.pone.0173339)
Supplement: S2 File — (DOCX) [file pone.0173339.s002.docx]

| **Section/topic** | **#** | **Checklist item** | **Reported on page #** |
| --- | --- | --- | --- |
| **TITLE** | | |  |
| Title | 1 | Gene-set meta-analysis of lung cancer identifies pathway related to systemic lupus erythematosus |  |
| **ABSTRACT** | | |  |
| Structured summary | 2 | **Introduction:** Gene-set analysis (GSA) is an approach using the results of single-marker genome-wide association studies when investigating pathways as a whole with respect to the genetic basis of a disease. **Methods:** We performed a meta-analysis of seven GSAs for lung cancer, applying the method META-GSA. Overall, the information taken from 11,365 cases and 22,505 controls from within the TRICL/ILCCO consortia was used to investigate a total of 234 pathways from the Kyoto Encyclopedia of Genes and Genomes (KEGG) database. **Results:** META-GSA reveals the systemic lupus erythematosus KEGG pathway *hsa05322*, driven by the gene region 6p21-22, as also implicated in lung cancer (p=0.0306). This gene region is known to be associated with squamous cell lung carcinoma. The most important genes driving the significance of this pathway belong to the genomic areas *HIST1-H4L, -1BN, -2BN, -H2AK, -H4K* and *C2/C4A/C4B*. Within these areas, the markers most significantly associated with LC are rs13194781 (located within HIST12BN) and rs1270942 (located between *C2* and *C4A*). **Conclusions:** We have discovered a pathway currently marked as specific to systemic lupus erythematosus as being significantly implicated in lung cancer. The gene region 6p21-22 in this pathway appears to be more extensively associated with lung cancer than previously assumed. Given wide-stretched linkage disequilibrium to the area *APOM/BAG6/MSH5*, there is currently simply not enough information or evidence to conclude whether the potential pleiotropy of lung cancer and systemic lupus erythematosus is spurious, biological, or mediated. Further research into this pathway and gene region will be necessary. | 2 |
| **INTRODUCTION** | | |  |
| Rationale | 3 | The genetically susceptibility to Lung Cancer is complex. Multiple related genes may interact can be missed by singe marker association analyses. |  |
| Objectives | 4 | **Questions being addressed**: Identification of predefined sets of gens, being enriched across studies with genes associated to lung cancer at a nominal significance level, accounting for correlation across studies in the patterns resulting from single marker association point estimates  **Participants**: Lung cancer patients and hospital or population controls  **Intervention**: non  **Comparisons**: at the GSA level: “gene set of interest” with all “remaining genes” at the GWAS level: cases with controls  **Outcomes**: p-values  **Study design:** Meta-analysis of gene-set analyses (GSA) based on genome-wide association studies (GWASs). | 19-20 |
| **METHODS** | | |  |
| Protocol and registration | 5 | no review protocol exists, because all | 19 |
| Eligibility criteria | 6 | Already and newly performed GSA on GWAS were combined addressing lung cancer with at least 300 cases and 300 controls | 19 |
| Information sources | 7 | Data shared within the *International Lung Cancer Consortium (ILCCO)* | 19 |
| Search | 8 | -- |  |
| Study selection | 9 | -- |  |
| Data collection process | 10 | Four studies had previously been used to compare the performance of four different GSA approaches by Fehringer Get al. (2012) PLoS One 7: e31816. Three further Studies were made available for this meta-analysis by members of the ILCCO. | 19 |
| Data items | 11 | GWAS: odds ratio and p-value for single marker association test , snp-to-gene allocation;  GSA, p-values and gene-to-pathway allocation |  |
| Risk of bias in individual studies | 12 | GWAS: any remaining risk of case-control studies, after careful accomplishment GSA: insufficient marker coverage and insufficient calculation of gene-level statistics |  |
| Summary measures | 13 | p-value |  |
| Synthesis of results | 14 | described in detail in the manuscript, an a previous publications (Rosenberger et al., META-GSA: Combining Findings from Gene-Set Analyses across Several Genome-Wide Association Studies, PLoS One. 2015 Oct 26;10(10):e0140179. doi: 10.1371/journal.pone.0140179. eCollection 2015.) | 21-23 |

Page 1 of 2

| **Section/topic** | **#** | **Checklist item** | **Reported on page #** |
| --- | --- | --- | --- |
| Risk of bias across studies | 15 | restricted study selection one of the four studies investigated small cell lung cancer patients only another of the four studies focused on young cases and controls (<50 years of age) |  |
| Additional analyses | 16 | -- |  |
| **RESULTS** | | |  |
| Study selection | 17 | -- |  |
| Study characteristics | 18 | Study characteristics are given in detail in: Fehringer G, Liu G, Briollais L, Brennan P, Amos CI, et al. (2012) Comparison of Pathway Analysis Approaches Using Lung Cancer GWAS Data Sets. PLoS One 7: e31816 and in Table 1 | 20 |
| Risk of bias within studies | 19 | -- |  |
| Results of individual studies | 20 | -- |  |
| Synthesis of results | 21 | The KEGG pathway *hsa05322 (*systemic lupus erythematosus) is significantly implicated in lung cancer. The gene region 6p21-22 in this pathway appears to be more extensively associated with lung cancer than previously assumed. | 22,34 |
| Risk of bias across studies | 22 | -- |  |
| Additional analysis | 23 | -- |  |
| **DISCUSSION** | | |  |
| Summary of evidence | 24 | -- |  |
| Limitations | 25 | restricted study selection |  |
| Conclusions | 26 | -- |  |
| **FUNDING** | | |  |
| Funding | 27 | The investigations in this article were supported by the grant from the National Institute of Health (NIH) (U19CA148127). The funding has no impact onto the results. |  |

Page 2 of 2
